# The Institutional Library

**Published:** 1919-12-06

**Authors:** 


### THE INSTITUTIONAL LIBRARY.

### THE GREAT WAR AND THE R.A.M.C.*

THE work of the Royal Army Medical Corps during the weary years of the trench war became constantly more standardised, more specialised, and indubitably more efficient. The war of positions meant, amongst other things, that hospital arrangements, always necessarily makeshift affairs during a war of rapid manœuvre, became ever more complete; that lines of evacuation of the wounded and sick became ever more speedy and more comfortable; and that, in short, the whole medical machine could and did run more smoothly. It is this swift, orderly, comfortable handling of the casualties that most of our soldiers experienced who went through the hands of the medical services. But it is as well not to forget that in the beginning things were far otherwise; and in this volume, the first of a popular medical history of the war, Colonel Brereton shows how and why the medical services struggled against heavy odds in their endeavour to do their duty by their combatant comrades. The book takes the tale only as far as the move from the Aisne to Flanders, early in October 1914; but it is packed with interest and with incident from first to last. At this rate, a score or more of volumes will be required to complete the series; but more probably it will be found expedient to deal less with the exact movements of individual medical units in succeeding volumes, and more with general principles of development, illustrated by incidents of the work of the R.A.M.C. chosen as typical of the whole.

Although it is true that, judged by the subsequent expansion of our armies and those of our allies and our enemies, the retreat from Mons by our forces was hardly more than a rearguard action, yet it will probably continue to exercise a glamour over the British public in excess of its purely material importance. After all, Foch has shown us that *moral* is all important in war; and it cannot be doubted that the fine fighting spirit of the five divisions that took part in those actions, together with the historic records they stood for and the traditions of the "old Army" which they upheld, put heart into our new armies and enabled the British people to start the long strain of the war on good terms with itself. That the R.A.M.C. was worthy to take its place among the "Contemptibles" no one doubts; at least, if such there be, he may be recommended to read Colonel Brereton's book. The story he unfolds is not one of unchequered success, but it at least is one of unabated effort. Had Sir John French's two Corps been provided with motor ambulances, the R.A.M.C. would have saved far more of the wounded than they did; and those wounded would have been saved tortures in many cases too terrible to describe. The fault lay not with the medical service, which had pressed again and again for these ambulances before the war, but with the purblind politicians who led the country straight into war without having taken the most essential precautions to be ready for it.

It is, perhaps, in the handling of incidents that the author is at his best, due it may be supposed to his long experience as a writer of fiction, which is often not so strange as truth, especially in war. The account of the adventures of the 4th and 19th Field Ambulances, which resulted in the capture of sixteen officers and over two hundred men of the R.A.M.C. at Landrecies after the famous fight there, does not make it clear who was responsible for this piece of stupidity: this is only one instance of Colonel Brereton's discreet silence about incidents of which he must know more than he tells. His reminder in connection with this incident that, whereas by the Geneva Convention all these officers and men should have been set free, the Germans deliberately and without excuse kept the whole lot prisoners for many months, is timely: the public memory of England is all too short over breaches of faith by our enemies, and it is right and proper that this particular one should be emphasised. The operations on the Aisne, or rather the work of the R.A.M.C. in connection therewith, are especially well described in a series of graphic pen pictures. Alike for general grasp of many sides of a large and complicated subject, for his powers of descriptive writing, and for his ability in presenting technicalities so as to be understanded of the people, the author has shown his fitness for the task he has undertaken; and his following volumes will be looked forward to with great interest.

* *The Great War and the R.A.M.C.* By Lieut.-Colonel F. S. BRERETON, R.A.M.C. (Constable and Co. 1919. Pp. 300. Price 12s. 6d. net.)

**The Redemption of Religion.** By CHARLES GARDNER. (London: Longmans, Green and Co. Price 7s. 6d. net.)

HOSPITAL chaplains, particularly those who have been forced to take their work seriously in the war hospitals at home, must often have wished for a guide to the general results and effects of the Higher Criticism. No cleric worthy of his ordination can ignore that criticism. It is his duty to digest it so that he may extract therefrom its contribution to the Faith. For criticism of its nature is contributive; the more learned the subject the more contributive is criticism likely to be; and he who will learn nothing from his opponents is punished by becoming incapable of learning from his friends. "The Redemption of Religion," as Mr. Gardner explains in his scholarly preface, is an attempt to extract what is valuable in the Higher Criticism, since "some of us believe that the Higher Criticism has a positive value if only one can get at it." The time is ripe for such a book, and our respect goes forth to the author for attempting to supply it. He begins with two great historical pictures which describe the meeting of Abelard with St. Bernard in 1139, and of Luther with the Diet of Worms in 1521. After tracing the relation of the rational spirit to the Church from Abelard to Luther and from Luther to our own day, the writer then devotes two chapters to "The Jesus of

**The Institutional Library**—(*continued*).

Criticism." In these he attempts to describe "what is the residuum to which many critics would consent." The attempt is of great interest, but its praiseworthy endeavour to describe nothing but that residuum necessarily leaves the reader in the dark as to the precise contributions which different critics have provided, as to Mr. Gardner's reasons for his selections and rejections, and as to the extent to which his conclusions are likely to command critical assent. Mr. Gardner's method, chosen no doubt for the sake of clearness and brevity, places us entirely in his hands; but, in the absence of direct evidence of his value as a guide, there is a mass of indirect evidence (not only in these pages, but in his other books) to his distinguished general claims upon our attention. The witness of the New Testament and the meaning to be attached to Jesus' teaching of the Kingdom are then described by reference to the Apocalyptic literature, in which the utterances of Jesus show Him to have been steeped. The Master's own claims, the attitude of St. Paul and of the Church to His Person are discussed, and we are then presented with the Christ "of experience." The Atonement and the Ascension and Resurrection are treated in two chapters, wherein their subjective and historical truth are severally considered. The last three chapters examine the relation of the visible Church to the Kingdom, give the Master's and the Church's teaching on escatology and eternal life, and discuss the process by which these teachings have assumed their form, and how that form must be regarded in relation to the New Testament. Such in brief is the skeleton of the book; but no summary can convey the *élan* of its style, the author's fire and learning, or the sense which he gives of caring for nothing but the truth, wherever that guiding star shall lead him. Rationalist and Christian will find that he is, considered controversially, of neither party, except in so far as his conclusions suggest that there is more substance in theological Christianity than most of its professors or opponents are aware. The book pulses with life, beside being learned and considerate; but its effect would have been heightened if Mr. Gardner had paid a little more attention to the task of removing small awkwardnesses of phrase ("are each," instead of are severally, on p. viii., is one example), which are annoying in a conscientious writer of his standing. The three final chapters are brilliant in exposition, alive with learning, sober with thought. Every hospital chaplain ought to regard the work as a rare handbook. It deserves a place in every institutional library.

**The Natural History of the Child.** By Dr. Courtenay Dunn. (London: Sampson Low, Marston and Co. 1919. Pp. 319.)

The author of this book is a keen student as well as a lover of children; and has a fine bevy of his own to boot, judging by the frontispiece! He apparently keeps a commonplace book in which he enters all sorts of quaint and curious jottings concerning childhood, and has now classified these and published them. He can tell you the age of the youngest child ever judicially executed in England, the youngest ever married, the youngest ever created a cardinal, and a great many other interesting things. He has a sharp eye for a *jeu d'esprit*, or a clever poem, and a happy knack of quoting them. At the same time he is, it is to be feared, not invariably trustworthy over technical matters. The "explanation" given of the alleged fact that a child prefers to look at a picture upside-down is an extraordinary example of muddled thought in a member of a learned profession. Nor can it be agreed that "eye trouble" in later life is due to over-exposure to light during the first days of a baby's existence. When he makes the astonishing statement that "diphtheria in puberty is really a dangerous disease," one is reminded of the fact that it is unlucky to break one's neck upon a Friday. In the chapter on schools, which is full of interesting miscellanea, Dr. Dunn states that at Stoneyhurst College (the well-known Roman Catholic school) holidays are known in the school slang as "Blandykes." This is an imported word, says Dr. Dunn, from St. Omer, where at the Roman Catholic seminary the boys are allowed on holidays to go to a neighbouring place called Blandyke. This latter place is doubtless the village of Blendecques, headquarters of the Second Army for a very long time during the war. Without being too positive, we doubt whether the "decques" of this name means "dyke," though the whole country is very much intersected by water lanes; for there are other place names in the district—*e.g.* Eperlecques—which seem to have no possible connection with "dykes."

**Menders of the Maimed.** By Arthur Keith, F.R.C.S., F.R.S. (London: Henry Frowde, and Hodder and Stoughton. 1919. Pp. 335.)

Professor Keith has in view, judging by his preface, an educative effect upon the medical profession in the rudiments of modern orthopædic surgery. Such an effect is much needed, for orthopædic surgery is still, if less so than formerly, a subject to which the medical schools pay insufficient attention; it is one of several important branches of clinical study which have been crowded out of the curriculum by the undue calls upon the student's time unfortunately conceded to various "preliminary" sciences. The attempt deserves to succeed, and will succeed if the medical profession can be induced to read this book carefully. The powder is amply concealed by the jam, by which is implied that the educative aim is not obtruded, but is achieved by making every chapter one of general as well as professional interest; but it is there, and will make its beneficial presence felt later, as is the manner of powders in jam. Nor is it only the medical profession that stands to profit by Professor Keith's labours; the intelligent public at large could do worse than absorb his teachings, more especially his words of wisdom on the subject of "bone-setting."

The method adopted is a sequence of biographical sketches of prominent orthopædic surgeons, especially those who have made the most striking original contributions to the progress of that science. Hunter, Hilton, Owen Thomas, Arbuthnot Lane, Sayre, Stromeyer, Syme, Wolff, and others are in this band. After that various subjects, such as bone grafting and tendon transplanting, are dealt with, and the history of their discovery and progress is reported. The method has the great advantage of being full of human interest (the "personal touch"), and of compelling the closest attention to first principles rather than to details. Some of the portraits of the eminent surgeons mentioned are not very good, or are poorly reproduced. Sir W. Arbuthnot Lane is a case in point, and there are others to whom the same criticism applies. A good index is supplied to this very interesting and instructive book.

**Mothercraft.** Selection from Courses of Lectures on Infant Care. Third Edition, revised and enlarged. (London: The National League for Health, Maternity, and Child Welfare. 1919. Price 5s.)

This book now combines theoretical and practical information upon a subject ever increasing and widening its boundaries with instruction to the student and interest to the lay mind. The chapters dealing with the subject of health laws and the law in relation to child welfare, and the appendix, including the examiners' questions and notes, should appeal to all students of this subject by their clear, concise exposition.

